# Relationships between orthostatic hypotension, frailty, falling and mortality in elderly care home residents

**DOI:** 10.1186/s12877-019-1082-6

**Published:** 2019-03-13

**Authors:** Brett H. Shaw, Dave Borrel, Kimiya Sabbaghan, Colton Kum, Yijian Yang, Stephen N. Robinovitch, Victoria E. Claydon

**Affiliations:** Department of Biomedical Physiology and Kinesiology, Simon Fraser University, Burnaby, BC V5A 1S6 Canada

**Keywords:** Frailty, Orthostatic hypotension, Falling, Older adults

## Abstract

**Background:**

Orthostatic hypotension (OH; profound falls in blood pressure when upright) is a common deficit that increases in incidence with age, and may be associated with falling risk. Deficit accumulation results in frailty, regarded as enhanced vulnerability to adverse outcomes. We aimed to evaluate the relationships between OH, frailty, falling and mortality in elderly care home residents.

**Methods:**

From the Minimum Data Set (MDS) document, a frailty index (FI-MDS) was generated from a list of 58 deficits, ranging from 0 (no deficits) to 1.0 (58 deficits). OH was evaluated from beat-to-beat blood pressure and heart rate (finger plethysmography) collected during a 15-min supine-seated orthostatic stress test. Retrospective and prospective falling rates (falls/year) were extracted from facility falls incident reports. All-cause 3-year mortality was determined. Data are reported as mean ± standard error.

**Results:**

Data were obtained from 116 older adults (aged 84.2 ± 0.9 years; 44% males) living in two long term care facilities. The mean FI-MDS was 0.36 ± 0.01; FI-MDS was correlated with age (r = 0.277; *p* = 0.003). Those who were frail (FI ≥ 0.27) had larger Initial (− 17.8 ± 4.2 vs − 6.1 ± 3.3 mmHg, *p* = 0.03) and Consensus (− 22.7 ± 4.3 vs − 11.5 ± 3.3 mmHg, *p* = 0.04) orthostatic reductions in systolic arterial pressure. Frail individuals had higher prospective and retrospective falling rates and higher 3-year mortality. Receiver operating characteristic curves evaluated the ability of FI-MDS alone to predict prospective falls (sensitivity 72%, specificity 36%), Consensus OH (sensitivity 68%, specificity 60%) and 3-year mortality (sensitivity 77%, specificity 49%). Kaplan Meier survival analyses showed significantly higher 3-year mortality in those who were frail compared to the non-frail (*p* = 0.005).

**Conclusions:**

Frailty can be captured using a frailty index based on MDS data in elderly individuals living in long term care, and is related to susceptibility to orthostatic hypotension, falling risk and 3-year mortality. Use of the MDS to generate a frailty index may represent a simple and convenient risk assessment tool for older adults living in long term care. Older adults who are both frail and have impaired orthostatic blood pressure control have a particularly high risk of falling and should receive tailored management to mitigate this risk.

**Electronic supplementary material:**

The online version of this article (10.1186/s12877-019-1082-6) contains supplementary material, which is available to authorized users.

## Background

With advances in medical care, growing technology, and public health efforts, life expectancies continue to rise despite the accumulation of age-related deficits [1]. However, while the risk of adverse health outcomes increases with aging, there is considerable variation in risk, even among those with the same chronological age [2]. Frailty, which reflects multisystem physiological change and vulnerability to internal and external stressors, is a concept used to quantify this elevated risk of adverse health outcomes, and reflects the accumulation of individual deficits that cumulatively result in frailty [3]. Frailty is an evolving concept that is distinct from aging, disability and the presence of comorbidity [4]. It can be defined as a dynamic state affecting an individual, with losses experienced in one or more domains of human functioning, incorporating physical, psychological and social components, that adversely affect outcomes [5]. Due to the increased prevalence of frailty among older adults, the World Health Organization has recognized frailty as an emerging public health interest [6]. Since then, extensive research regarding the mechanisms, predictive ability, and clinical management of frailty have made it a valid tool for research [7, 8], and the development of standardised approaches to screening and risk assessment for frailty have been highlighted as a research priority [4]. Indeed, frailty indices have been shown to be important in guiding patient care [4, 7], and a survey of 356 Canadian healthcare professionals revealed that 69% considered frailty to be a clinically useful measure [8].

Operationalizing frailty is challenging and numerous indices have been proposed [3, 9–12]. Use of a frailty index (FI) is commonly employed because this validated measure draws from age-related deficits in multiple domains and does not require performance measures, unlike other phenotypic assessments that consider specific signs or symptoms of frailty through assessments of gait speed and handgrip strength [13, 14]. The FI also permits some flexibility in deficits considered, as long as at least 30–40 deficits are included, and provides a continuous scoring system with identifiable cut-points for frailty severity [3, 10, 15, 16]. Several frailty measures have been derived from standard assessment surveys such as the risk analysis index (RAI), and comprehensive geriatric assessment (CGA), both of which have been well validated [11, 17, 18]. In nursing homes in the US and Canada the minimum dataset (MDS) is a mandated process for clinical assessment of all residents, and provides a comprehensive assessment of functional capacity. This assessment encompasses multiple domains considered in frailty scores, and has been utilised to operationalise frailty in the acute care hospital setting, but has not been used for a full FI in the long term care setting [18]. A benefit to utilising an existing dataset such as the MDS to operationalise frailty, particularly in older adults in a long term care setting, is the avoidance of performance based tests or self-report of impairments, which are challenging in this cohort.

We are interested in the relationships between frailty and orthostatic hypotension (OH, excessive decreases in blood pressure when upright). OH often reflects autonomic nervous dysfunction, which may be associated with widespread alterations in physiological functioning, and therefore frailty [19]. In addition, many risk factors for OH (polypharmacy, supine hypertension, autonomic neuropathy and hypovolemia) would constitute deficits in frailty indices. Indeed OH has been defined as a sign of a final common pathway for various forms of disordered physiology, and an indicator of frailty [20, 21]. In community dwelling older adults, symptoms of orthostatic intolerance (dizziness, light-headedness or unsteadiness when standing up) were associated with markers of frailty [22]. However, no studies have examined the relationships between frailty and OH in older adults living in long term care facilities, or have used more sensitive measures to assess cardiovascular autonomic control.

In community dwelling older adults, frailty has been shown to be associated with falls, cognitive impairment, disability, hospitalisation and institutionalisation [4, 23], and is the most common chronic condition leading to death in older adults [24]. There may also be associations between impaired cardiovascular reflex control in community dwelling seniors and frailty [25, 26] and mortality [27]. Impaired orthostatic control of blood pressure and cerebral blood flow are risk factors for falling in older adults living in long term care [28], and OH may be related to impaired standing balance in older adults [29]. Indeed, 11% of video-captured falls in elderly residents in long-term care occurred due to collapse or loss of consciousness, with 41% due to incorrect transfer or shift of bodyweight [30]. The associations between OH, frailty, falls and mortality are of particular concern in older adults because one-third of individuals aged > 65 years fall at least once each year [31] and falls account for 85% of injury-related hospitalizations [32]. Older adults in residential care are at particular risk of falls and fall-related injury [33, 34] and have a high incidence of OH [35]. Given the potential interrelationships between frailty, OH, falling and mortality, in this study we aimed to: (i) develop a FI using a standardised data collection tool (MDS) to better evaluate frailty in older adults residing in long term care facilities; (ii) examine the relationships between frailty, OH and falling in older adults living in long term care facilities; (iii) evaluate the relative impact of frailty, OH and falling on mortality in older adults living in long term care facilities. The identification of those most at risk would enable targeted risk reduction.

## Methods

This study received ethical approval from the Office of Research Ethics at Simon Fraser University and conforms to the principles outlined in the Declaration of Helsinki. Written informed consent was obtained prior to participation from the participants or their legal designate (with verbal assent). Participants were recruited from two long-term care facilities. They were eligible to participate if they had resided in the facility for at least two years, were aged ≥65 years, and it was possible to obtain access to their Minimum Data Set (MDS). Data were extracted from the MDS and used to derive an index of frailty. Falling risk was determined from facility fall incidence reports. A randomly selected subset of individuals (*n* = 55) also completed a cardiovascular assessment [32] to determine susceptibility to OH. Participants were followed for 3 years from the date of assessment over which time their mortality was determined.

### Cardiovascular assessment

Participants underwent a passive seated orthostatic stress test according to our standard laboratory procedure [32, 36]. Participants remained in a supine position for 15 min prior to being passively moved to an upright-seated position for an additional 15 min of recordings. Beat-to-beat arterial blood pressure was continuously recorded throughout testing (Finometer® Pro, Finapres Medical Systems B.V., Amsterdam, The Netherlands). Heart rate and rhythm were monitored using a lead II electrocardiogram (ECG; Finapres ECG Module, Finapres Medical Systems, Amsterdam, The Netherlands). Data were sampled at 1 KHz using an analog:digital converter (Powerlab 16/30, AD Instruments, Colorado Springs, CO, USA). Cardiovascular assessments were conducted as close as possible in time to the date of completion of the MDS document. The mean time interval between the two measures was ±1.6 months.

Systolic (SAP), diastolic (DAP), and mean (MAP) arterial pressures were detected for each blood pressure waveform. Heart rate was also computed on a beat-to-beat basis. During the 15-min supine period, 30-s averages of all parameters were obtained to record a steady-state baseline value for each parameter. Responses to orthostasis were averaged over 5 s periods and were expressed both in absolute values, and as percentages relative to the supine value at the following time points: the lowest 5-s average in the upright phase (or highest for heart rate) within the first 30 s (*Initial);* first 3 min (*Consensus);* and from 3 to 15 min *(Delayed).* These hemodynamic intervals reflect clinically relevant time points for the varying definitions of OH [37]. We focused particularly on SAP responses because they are associated with a number of clinical markers, including all-cause mortality [26, 27, 38]. Consensus and Delayed OH were defined as a decrease in SAP ≥20 mmHg or in DAP ≥10 mmHg at each time point of interest [37]. Initial OH was defined in two ways: (i) a decrease in SAP ≥40 mmHg or in DAP ≥20 mmHg within the first 30 s [37]; (ii) as ≤80% recovery of SAP relative to the supine value at 60 s (*Recovery OH*) [39].

### Frailty

The Full Resident Assessment Instrument-Minimum Data Set (MDS) 2.0 is a standardized assessment used in all long-term care facilities across Canada and the USA. The full assessment is completed annually with a sub-assessment repeated every 3 months; over 30 countries are now collecting these data [40]. We used this document to derive a list of 58 deficits (Additional File 1) that can be used to generate a frailty index (FI-MDS) according to previously established principles [3, 10, 15, 16, 18]. Deficits were sub-grouped into 12 categories representing multiple functional domains (Additional File 2). Each deficit was assigned a 0 (absence of condition or attribute) or 1 (presence of condition or attribute) score. Body mass index was assigned a 0 score unless it was < 18.5 kg/m^2^ or ≥ 30 kg/m^2^, in which case a score of 1 was assigned. Medication usage was scored according to the number of medications used: > 5 = 1; > 10 = 2; > 15 = 3; > 20 = 4 [18]. Some variables in the frailty index that are concerned with the history of a disorder, and do not change over time, were derived from the full MDS assessment that is completed annually. The mean of the deficit scores was considered to be the frailty index, ranging from 0 (no deficits) to 1.0 (58 deficits).

### Falling

Participants’ falling susceptibility was ascertained through review of fall incident report forms from both facilities for the year preceding and the year following the date of the frailty score. As a requirement for accreditation in British Columbia, incident reports are completed for every fall event reported or witnessed within each facility [41]. For some analyses, participants were categorised as being either a faller (≥1 retrospective falls in the past year) or a non-faller (0 retrospective falls in the past year), as in previous studies [42, 43]. Similarly, prospective falling was determined for the year following the date of the frailty score. Six participants declined consent for falling data to be collected.

### Mortality

Participant all-cause mortality was determined at 36 months (3-year mortality) after the date of the initial assessment (defined as the date of completion of the MDS document or cardiovascular assessment). Survival rates were determined to the nearest month for all participants. Participant discharge to a higher level of care was also noted as an outcome.

### Statistical analyses

Unless stated otherwise, all values are reported as mean ± standard error of the mean. Level of significance was set at α = 0.05. Statistical analyses were performed using SigmaPlot version 11 (Systat Software Inc., San Jose, CA). Differences between subgroups were examined using unpaired Student’s t test or Mann-Whitney U test as appropriate, or Fisher’s exact test for categorical data. Correlations between variables were determined with the Pearson or Spearman test for parametric or non-parametric data respectively. For correlative analyses, the FI-MDS was log transformed because the distribution was bimodal. Multiple linear regression analyses were conducted to identify factors influencing prospective falling rates (the number of falls in the year following the frailty assessment) and 3-year mortality. The relationship between retrospective and prospective falling rates in frail and non-frail individuals was examined using a two-way repeated measures analysis of variance. We used receiver operating characteristic (ROC) curves to evaluate the sensitivity and specificity of the FI-MDS to predict future falls and presence of Consensus OH. The area under the ROC curve (AUC) was also calculated. Log-Rank Kaplan Meier Survival analyses were completed to determine the influence of frailty, falling and the presence of Consensus OH on mortality. Mortality and discharge to a higher level of care were both considered to be “events”.

## Results

### Participant demographics

**Fig. 1:**
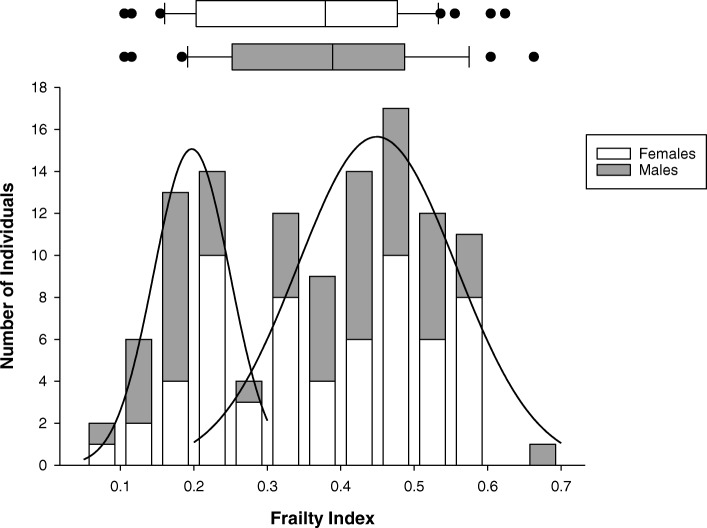
Distribution of FI-MDS for the cohort as a whole (n = 116). The density distribution of the FI-MDS was bimodal, with two subgroups of non-frail and frail individuals. The crossing point distinguishing the two sub groups was approximately FI-MDS = 0.27. The horizontal box plots represent the mean FI-MDS in males and females

We recruited 116 participants (mean age 84.2 ± 0.9 years, with 44% males) with a mean FI-MDS of 0.36 ± 0.01 (range 0.09–0.66) (Table 1). The distribution of frailty was bimodal, with a crossing point at a FI-MDS = 0.27 (Fig. 1). There were no significant sex differences in FI-MDS (female 0.37 ± 0.02; male 0.34 ± 0.02; *p* = 0.272) (Fig. 1). There was a significant positive correlation between the FI-MDS and age (r = 0.278; *p* = 0.003) (Table 2). Based on previous criteria [15] 12% of our cohort would be considered “non-frail”; 27% “pre-frail”; 52% “more frail”; 14% “frail” and 9% “most frail”. Overall, 64% of participants had experienced a fall in the previous year, with a mean fall rate of 2.36 ± 0.36 falls in the previous year (range 0–27 falls in the previous year). Those who had fallen previously were more likely to experience prospective falls (r = 0.549; *p* < 0.001) (Table 2). Cardiovascular data were obtained in a subset of 55 individuals. The prevalence of OH in these individuals was: Initial OH, 10%; Recovery OH, 6%; Consensus OH, 44%; Delayed OH, 49%. The overall incidence of OH, of any subtype, was 62%; 35% of individuals met criteria for more than one subtype of OH.

**Table 1:** Comparison between frail and non-frail individuals and between fallers and non-fallers (n = 116)

	All	Non-Frail	Frail	*p*	Non-Faller	Faller	*p*
N	116	37	79	–	40	70	–
FI-MDS	0.36 ± 0.01	**0.18 ± 0.01**	**0.44 ± 0.01**	**< 0.0001**	**0.31 ± 0.02**	**0.40 + 0.02**	**0.002**
Age (years)	84.2 ± 0.9	82.9 ± 1.7	84.8 ± 1.1	0.33	85.0 ± 1.3	84.7 ± 1.1	0.89
Male (%)	44.0	48.6	41.8	0.31	45.0	40.0	0.69
Retrospective Falls/Year	2.36 ± 0.36	**1.03 ± 0.3**	**2.94 ± 0.5**	**0.001**	**0 ± 0**	**3.71 ± 0.5**	**< 0.0001**
Prospective Falls/Year	3.26 ± 0.50	**1.94 ± 0.4**	**3.84 ± 0.7**	**0.02**	**1.71 ± 0.37**	**4.10 ± 0.73**	**0.004**
Supine SAP (mmHg)^a^	138.1 ± 3.4	138.9 ± 4.1	137.4 ± 5.4	0.83	137.2 ± 5.3	140.4 ± 5.1	0.66
Supine DAP (mmHg)^a^	69.3 ± 1.6	66.8 ± 2.5	71.5 ± 2.1	0.15	67.8 ± 2.4	71.0 ± 2.5	0.36
Initial HR response (bpm)^a^	6.7 ± 1.2	+ 5.5 ± 1.1	+ 8.2 ± 2.2	0.21	5.5 ± 1.1	7.9 ± 2.2	0.33
Recovery SAP (%)^a^	101.5 ± 2.1	*105.5 ± 2.9*	*97.9 ± 2.8*	*0.06*	104.3 ± 3.8	99.8 ± 2.3	0.31
Initial ∆SAP (mmHg)^a^	−12.30 ± 2.8	**−6.1 ± 3.3**	**−17.8 ± 4.2**	**0.03**	*−6.0 ± 4.0*	*−15.8 ± 3.9*	*0.08*
Consensus ∆SAP (mmHg)^a^	−17.2 ± 2.8	**−11.5 ± 3.3**	**−22.7 ± 4.3**	**0.04**	*−11.2 ± 3.9*	*− 21.5 ± 4.2*	*0.07*
Delayed ∆SAP (mmHg)^a^	−15.7 ± 2.44	−14.4 ± 2.0	−16.9 ± 4.4	0.61	−12.4 ± 3.7	−19.4 ± 3.7	0.19
3-year Mortality (months)	22.6 ± 1.1	**27.1 ± 1.9**	**20.5 ± 1.3**	**0.006**	21.8 ± 1.9	23.1 ± 1.4	0.57

Abbreviations: *SAP* systolic arterial pressure, *DAP* diastolic arterial pressure, *HR* heart rate, FI-MDS, minimum data set derived frailty index. ^a^sample size for these variables, n = 55 (non-frail *n* = 25; frail *n* = 30; non-faller *n* = 21; faller *n* = 34). Bold data indicate statistically significant differences. Italicised data indicate differences that did not quite achieve statistical significance

Frail individuals had higher retrospective and prospective falling rates, larger initial and consensus declines in systolic arterial pressure, and higher 3-year mortality than non-frail individuals. Retrospective fallers were more frail and had higher prospective falling rates than retrospective non-fallers. The outcome of mortality was considered met in participants who had died after 36 months (n = 69) or who had been discharged to a higher level of care (n = 6)

**Table 2:** Correlations between frailty, falling, markers of impaired blood pressure control and mortality (n = 116)

	Frailty (FI-MDS)		Prospective Falls/Year	
	r	*p*	r	*P*
Frailty (FI-MDS)	–	–	**0.205**	**0.033**
Age (years)	**0.278**	**0.003**	0.068	0.485
Retrospective Falls/Year	**0.302**	**0.001**	**0.549**	**< 0.00001**
Prospective Falls/Year	**0.205**	**0.033**	–	–
Initial Nadir SAP (mmHg)^a^	**−0.296**	**0.037**	*−0.274*	*0.072*
Consensus Nadir SAP (mmHg)^a^	*−0.235*	*0.097*	*−0.276*	*0.069*
Delayed Nadir SAP (mmHg)^a^	−0.127	0.373	**−0.302**	**0.046**
Recovery SAP (mmHg)^a^	−0.222	0.130	**−0.354**	**0.022**
Recovery SAP (%)^a^	−0.198	0.177	−0.229	0.145
3-year Mortality (months)	**−0.282**	**0.003**	0.035	0.719

Abbreviations: *SAP* systolic arterial pressure, *FI-MDS* minimum data set derived frailty index. ^a^sample size for these variables, *n* = 55. Bold data indicate statistically significant differences. Italicised data indicate differences that did not quite achieve statistical significance

Frailty was correlated with both retrospective and prospective falling rates, the initial orthostatic decrease in blood pressure, and 3-year mortality. The prospective falling rate was correlated with the retrospective falling rate, the magnitude of the orthostatic decrease in blood pressure, and the initial recovery in blood pressure. The outcome of mortality was considered met in participants who had died after 36 months (n = 69) or who had been discharged to a higher level of care (n = 6)

### Comparisons between frail and non-frail sub-groups

**Fig. 2:**
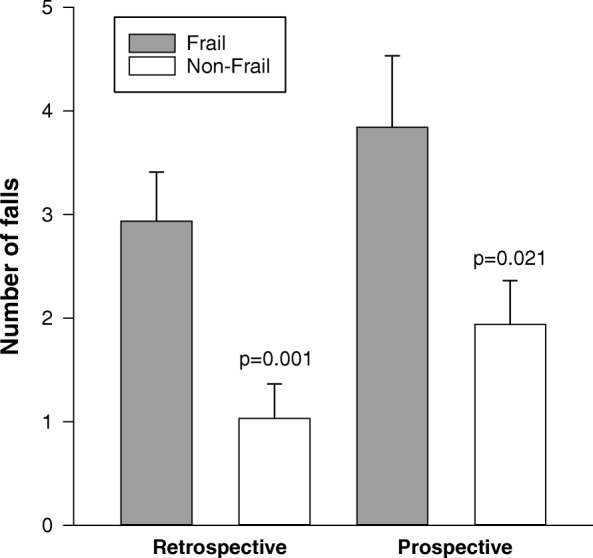
Retrospective and prospective falling rates in individuals who were frail and non-frail (n = 116). Those who were frail had higher retrospective and prospective falling rates (falls/year) than those who were non-frail

**Fig. 3:**
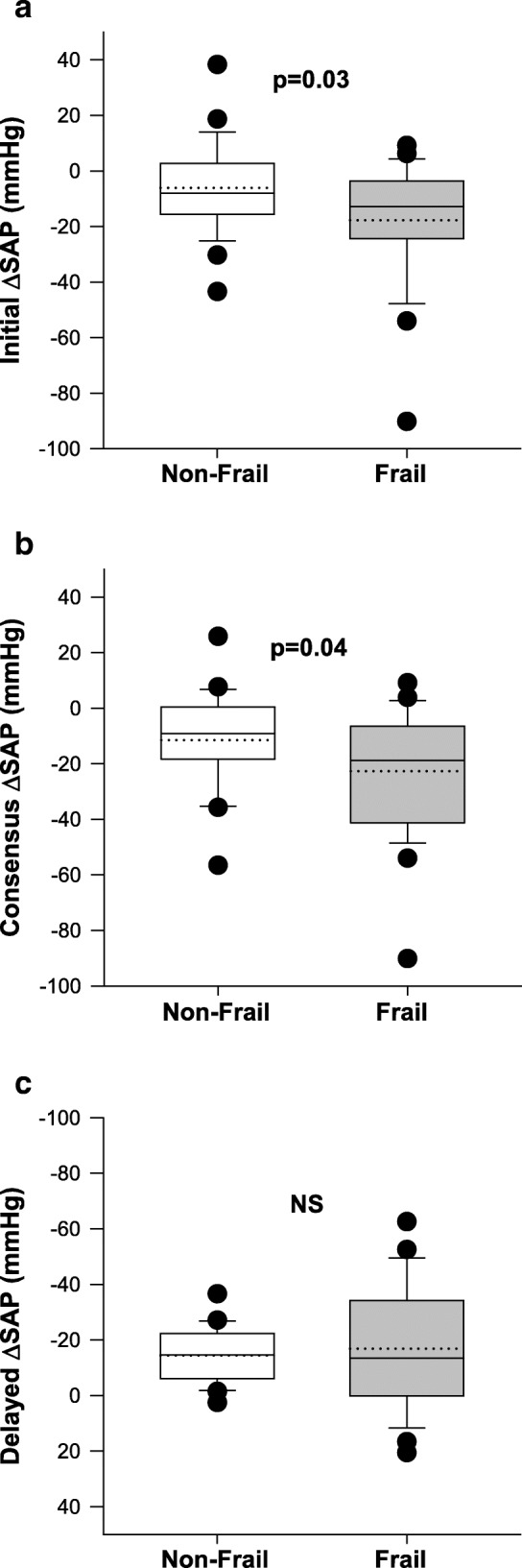
*Severity of orthostatic hypotension in frail and non-frail individuals (n = 55).* Those who were frail had larger Initial (**a**), and Consensus (**b**) declines in SAP than those who were non-frail. The delayed decline in SAP (**c**) was not different between groups. Solid horizontal lines indicate the median and dotted horizontal lines represent the mean. Abbreviations: *SAP* systolic arterial pressure, *NS* not significant

Participants were subdivided into two groups based on the bimodal distribution of frailty in this cohort, and classified as non-frail (FI < 0.27; *n* = 37) and frail (FI ≥ 0.27; *n* = 79) (Table 1). Individuals who were frail had significantly higher retrospective and prospective falling rates than those who were non-frail (Table 1 and Fig. 2). Frail individuals also had higher 3-year mortality than those who were non-frail (Table 1); there was a significant correlation between frailty and 3-year mortality (r = − 0.282; *p* = 0.003) (Table 2). Supine blood pressures and the heart rate responses to orthostasis were similar in the two groups (Table 1). Those who were frail had larger initial and Consensus declines in systolic arterial pressures than those who were non-frail (Table 1 and Fig. 3). The percentage recovery of the initial decrease in SAP tended to be impaired in frail individuals compared to non-frail individuals, but this did not quite reach statistical significance (*p* = 0.06). In frail individuals the prevalence of Initial OH, Recovery OH, Consensus OH and Delayed OH was 15, 12, 56 and 49%. In non-frail individuals the prevalence of Initial OH, Recovery OH, Consensus OH and Delayed OH was 4, 0, 32 and 48%. The magnitude of the initial decrease in SAP was negatively correlated with frailty (Table 2). The relationship between frailty and the Consensus decrease in SAP did not quite achieve statistical significance (*p* = 0.097).

**Fig. 4:**
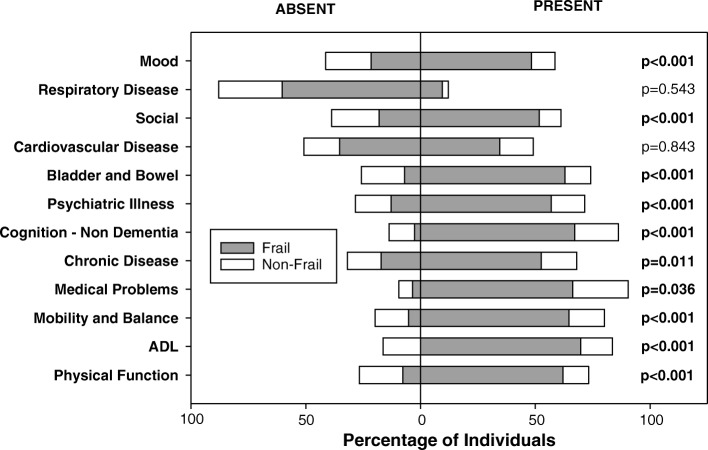
*Proportion of frail and non-frail individuals within each subdomain of deficits reported in the FI-MDS (n = 116).* Individuals were considered to be frail when their frailty index (FI-MDS) was ≥0.27. For a full description of the deficits considered within each subdomain see Additional File 2. Abbreviations: ADL, activities of daily living

We considered whether the subdomains from which the frailty scores were comprised were different between those who were frail and those who were non-frail (Fig. 4). There was a higher prevalence of deficits in each of the subdomains considered in the frail than the non-frail individuals, with the exception of cardiovascular disease and respiratory disease, which were equally prevalent in both frail and non-frail participants.

**Fig. 5:**
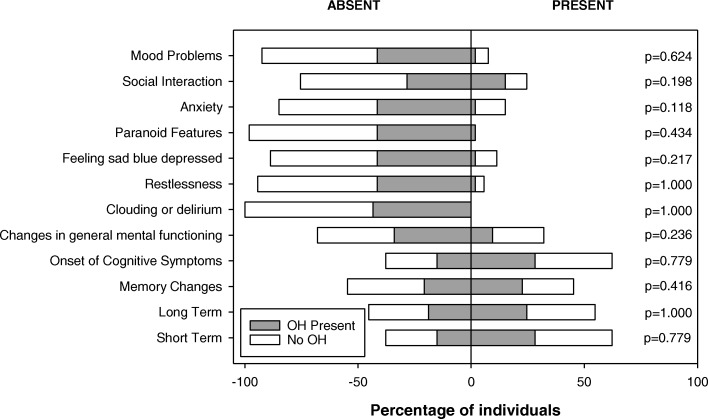
*Frequency of Consensus orthostatic hypotension (OH) among each cognitive symptom reported in the FI-MDS (n = 55).* Individuals were considered to have Consensus OH when there was decrease in SAP ≥20 mmHg or in DAP ≥10 mmHg within the first 3 min of being upright. Abbreviations: *OH* orthostatic hypotension

Given that OH manifests with reduced cerebral perfusion as a consequence of hypotension, we considered whether the cognitive symptoms considered were different between those with and without OH (Fig. 5). There were no significant differences in cognitive symptoms between those with and without OH. However, there was a higher incidence of admission to an Alzheimer’s or dementia special care unit among those who were frail (49%) compared to those who were not (16%) (*p* = 0.0009). There was a higher incidence of admission to an Alzheimer’s or dementia special care unit among retrospective fallers (43%) compared to non-fallers (23%) (*p* = 0.039).

### Relationships with retrospective falling risk

Those who had fallen in the previous year were more frail (FI-MDS: 0.40 ± 0.02) than those who had not fallen (FI-MDS: 0.31 ± 0.02; *p* < 0.01) (Table 1) and the prevalence of frailty was greater among individuals who had previously fallen (79%) compared to those who were not fallers (55%) (*p* = 0.0165). Those who had fallen in the previous year were more likely to fall in the following year than those who had not experienced a prior fall (mean prospective falling rate: prior fallers 4.10 ± 0.73 and prior non-fallers 1.71 ± 0.37 falls respectively, *p* = 0.004); there was a significant correlation between retrospective and prospective falling rates (r = 0.549, *p* < 0.00001) (Table 2). Those who had fallen previously tended to have larger Initial (non-fallers − 6.0 ± 4.0 mmHg; fallers − 15.8 ± 3.9; *p* = 0.08) and Consensus (non-fallers − 11.2 ± 3.9 mmHg; fallers − 21.5 ± 4.2 mmHg; *p* = 0.07) declines in systolic blood pressure compared to non-fallers, although this did not quite achieve statistical significance. In fallers the prevalence of Initial OH, Recovery OH, Consensus OH and Delayed OH was 12, 9, 54, and 46%. In non-fallers the prevalence of Initial OH, Recovery OH, Consensus OH and Delayed OH was 5, 5, 45, and 38%.

### Relationships with prospective falling risk

There were significant positive correlations between frailty and both retrospective (r = 0.302, *p* < 0.001) and prospective falling risk (r = 0.205, *p* = 0.03) (Table 2). The severity of the orthostatic reduction in SAP was also significantly correlated with prospective falling at the Delayed time-point (r = − 0.302, *p* = 0.046), and almost reached statistical significance for Initial (*p* = 0.072) and Consensus (*p* = 0.069) time points (Table 2**)**. The blood pressure at the 1-min recovery time-point was also correlated with the prospective falling risk (r = − 0.354, *p* = 0.022). Multiple regression analyses revealed that prospective falling could be predicted from a linear combination of FI-MDS and retrospective falling incidence (r = 0.550, p < 0.001). Prediction of prospective falling was enhanced when the recovery SAP was included in the model (r = 0.718, p < 0.001). The likelihood an individual would go on to fall prospectively was predicted with a sensitivity of 93% and specificity of 38% (prospective falling rate = 10.792-(0.0671 x SAP 1 min) + (0.802 x FI-MDS) + (1.233 x number of falls in the past year)).

### Frailty as a predictor of consensus OH and prospective falling risk

We considered whether the presence of frailty (based on the FI-MDS ≥0.27) was a predictor of prospective falling risk, or the presence of Consensus OH (the most commonly reported clinical subtype of OH). Receiver operating characteristic (ROC) curves were determined.

The presence of Consensus OH was predicted by frailty with 68% sensitivity and 60% specificity. Use of a more stringent cut-off for frailty (FI-MDS ≥ 3.0) did not improve the ability to predict the presence of Consensus OH from the FI-MDS (67% sensitivity and 58% specificity).

The risk of prospective falls (≥ 1 fall in the subsequent year) was predicted by frailty with 72% sensitivity and 36% specificity. We also considered whether the FI-MDS would better identify those at risk of recurrent falls, defined as ≥3 falls in the subsequent year (AUC: 0.586; sensitivity 80%; specificity 37%) or as ≥5 falls in the subsequent year (AUC: 0.643; sensitivity 83%; specificity 34%). Use of a more stringent cut off (FI-MDS ≥ 3.0) provided 83% sensitivity and 42% specificity to identify those at risk of recurrent falls (≥ 5 falls) in the subsequent year.

### Frailty, OH, falling and 3-year mortality

We considered the impact of frailty, falling and OH on 3-year mortality. The mortality end-point was considered to have been met where the participant died or was discharged to a higher level of care.

**Fig. 6:**
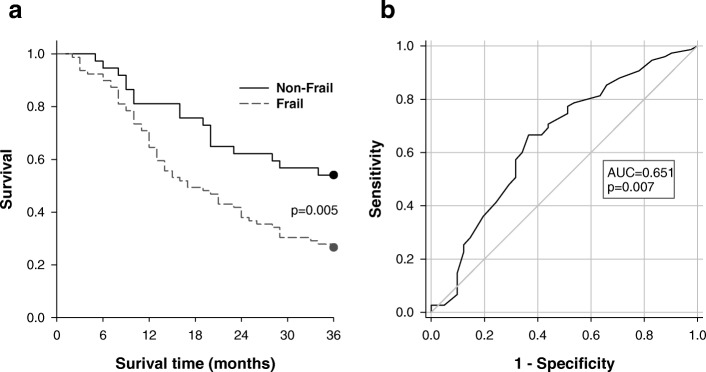
Relationships between frailty and 3-year mortality. (**a**) Kaplan-Meier survival analyses showing the impact of frailty (FI-MDS) on mortality (n = 116). The outcome of mortality was considered met in participants who had died after 36 months (*n* = 69) or who had been discharged to a higher level of care (n = 6). Individuals who were frail (FI-MDS ≥0.27) had a significantly higher 3-year mortality (*P* < 0.005) than those who were non-frail. (**b**) Receiver Operating Characteristic (ROC) curve for the prediction of 3-year mortality (n = 116) from the FI-MDS. The area under the curve (AUC) was 0.651 (*p* = 0.007) with 77% sensitivity and 49% specificity to predict 3-year mortality based on a FI-MDS ≥ 0.27

At the 3-year time point, 69 (60%) participants had died and 6 (5%) had been discharged to a higher level of care, with 41 (35%) participants alive and still resident in the long-term care facility. Individuals who were frail (FI ≥ 0.27) had a higher mortality (20.5 ± 1.3 months) than those were not frail (27.1 ± 1.9 months; *p* = 0.006). There were no significant differences in mortality between those with and without Consensus OH, or fallers (≥ 1 fall in the previous year) and non-fallers. Kaplan-Meier survival analyses revealed significantly higher 3-year mortality in individuals who were frail (*p* = 0.005) (Fig. 6). We constructed receiver operating characteristic (ROC) curves to evaluate the ability to predict 3-year mortality from the frailty index (Fig. 6). The presence of frailty (FI-MDS ≥0.27) was a significant predictor of mortality with a sensitivity of 77% and specificity of 49% (AUC = 0.651; *p* = 0.007).

Multiple regression analyses examining the relative contributions of Consensus OH, frailty and retrospective falling on 3-year mortality (R = 0.443, *p* = 0.013) identified a significant contribution from frailty (β = − 11.5 ± 3.6, *p* = 0.002). The contribution from Consensus OH did not quite achieve significance (β = 6.0 ± 3.3, *p* = 0.076). Falling status was not a significant determinant of 3-year mortality (β = 5.8 ± 3.4, *p* = 0.102).

## Discussion

We have demonstrated that frailty can be captured using a validated frailty index generated using MDS data in elderly individuals living in long term care, and is related to orthostatic cardiovascular control, falling risk and all-cause mortality. Frail individuals had larger Initial and Consensus orthostatic reductions in SAP, with impaired blood pressure recovery, a higher prevalence of OH, and greater mortality. Frail individuals also experienced higher prospective and retrospective rates of falling than the non-frail. Multiple regression analyses predicted prospective falling based on the frailty score and retrospective falling rates. The presence of OH and risk of future falls could be determined from the FI-MDS with high sensitivity (but poor specificity). The FI-MDS was also predictive of all-cause 3-year mortality.

### Use of the MDS to generate a frailty index

We used data obtained from the MDS to generate the frailty index in a manner consistent with a previous frailty index generated from the MDS in older adults admitted to acute care hospitals [18]. The benefits of this approach are that the frailty index could be derived from readily available data that is collected and updated consistently in long term care facilities without additional testing or scoring. In addition, in this older adult care home population there was a high incidence of individuals with mobility impairment and cognitive impairment, who may have found additional testing or questioning to evaluate phenotypic frailty prohibitively challenging. We considered whether use of a frailty index in an elderly care home population would be susceptible to a ceiling effect, but this was not the case, further highlighting the utility of this approach. As in previous studies, the FI we used assigned individual deficit scores using dichotomised variables [3]. Despite concerns that this may mitigate reliability and validity of responses [40], this approach has been extensively validated previously [11, 17]. Although use of the MDS document to generate a frailty score in the long term care setting has not been previously validated, the concept of a frailty index based on dichotomised variables from a variety of deficits is not new, and has been extensively validated previously [3, 18, 43]. Furthermore, use of a frailty index derived from the MDS has been validated in the acute care hospital setting [18]. This provides confidence in our use of an MDS-based frailty index in older adults in long term care.

In frail individuals, the greater deficit accumulation that they exhibited encompassed all domains considered, with the exception of respiratory disease and cardiovascular disease, which were equally distributed between the frail and non-frail. This may reflect the high general incidence of cardiovascular and respiratory disorders in older adults living in residential care, and suggests that the presence of cardiovascular or respiratory disease in older adults does not necessarily convey a risk of frailty in the absence of deficits in other domains.

We found a higher incidence of dementia among those who were frail, and this is consistent with previous literature [10, 44]. In frailty evaluations, mild cognitive impairments are often considered separately from severe dementia as the latter usually involves more disability, poorer physical performance and behavioural problems [44]. Given their close association, the consideration of cognitive impairment separately to dementia is important to avoid the FI becoming an indicator of dementia, and this was the approach taken in the present study. In addition to the association between dementia and frailty, we also found a higher incidence of dementia in those who had previously fallen than those who had not fallen. This may reflect a higher risk for falls in those with dementia, and/or an indirect association mediated by the higher incidence of frailty in prior fallers and those with dementia [45].

Similar to previous literature [2], frailty was positively associated with age. However, unlike in previous reports, it was not related to sex [44, 46]. This may reflect the demographics of older adults residing in long term care, which showed a predominance of females and a high proportion of frail individuals. Since those in a care home setting may have more deficits in general compared to community dwelling counterparts, sex differences in FI may be lost. In addition to population-based differences, methodological differences in the generation of the FI and scoring cut-point may also contribute to the ability to identify sex differences in frailty.

In this older institutionalised population, the frailty score was higher (FI 0.36 ± 0.01, aged 84 ± 0.9 years) than in a previous report of a vast sample of Canadian community dwelling seniors (FI 0.16 ± 0.1, aged 75 years) [15] but similar to other FI derived from long term care populations (FI 0.35 ± 0.1, aged 86 ± 0.2 years [47]; FI 0.35 ± 0.1, aged 88 ± 0.3 years [44]) and older adults admitted to acute care hospitals (FI 0.32 ± 0.1, aged 81 ± 0.2 [18]. Accordingly, we used a higher cut-point to distinguish the frail and non-frail in the present study, compared to one recommended cut point of 0.21 [15]. We defined the cut-point for frailty as 0.27 because this was the intersection point of the bimodal distribution of the FI-MDS in this cohort of older adults living in long term care. This approach was also used in a previous report which used the distributions of frail and non-frail individuals to derive an approximate frailty cut-point of 0.25 in community dwelling older adults [48]. Given that older adults in long term care are more frail than their community dwelling counterparts, a higher cut-off is needed to differentiate frail and non-frail individuals within a cohort with higher deficit accumulation. It is also important to note the wide range of FI-MDS values in the present study, which demonstrates the utility of this approach to stratify frailty, even in older adults resident in long term care, and was necessary to be able to detect associations between frailty and cardiovascular or falling outcomes.

### Relationships between frailty and orthostatic hypotension

We showed that individuals who were frail had larger Initial and Consensus declines in blood pressure, and impaired blood pressure recovery, upon the assumption of a seated posture. These findings are important because OH is known to be associated with considerable decrements in morbidity and mortality [18, 49] as well as cognitive decline [50]. Indeed, the severity of Consensus OH tended to be associated with 3-year mortality (*p* = 0.076) in the present study. We and others have previously described an increased risk of falling in individuals with OH, perhaps mediated by impaired cerebral perfusion [32] and this has a devastating impact, particularly in the elderly, in whom fall-associated injuries can limit independence and initiate terminal decline.

Our findings are compatible with previous reports in community-dwelling older adults that showed impaired initial orthostatic blood pressure control in those with increasing severity of frailty [2], which was identified as an independent predictor for falls [39]. Indeed, we showed that when stratified according to prior falling susceptibility, those with a history of falls tended to have more severe Initial and Consensus OH. The extension of these observations to older adults residing in long-term care further highlights the importance of considering frailty and OH as risk factors for falls, and implementation of management approaches that are tailored to those most at risk.

A previous study showed that a higher FI was associated with more symptoms of orthostatic intolerance in community dwelling participants [22]. However, unlike in the present study, they did not find an association between FI and objective measures of blood pressure, perhaps reflecting the different blood pressure measurement approaches employed. In the current study we used continuous beat-to-beat blood pressure instead of intermittent monitoring, better enabling identification of orthostatic blood pressure changes and therefore revealing greater susceptibility to OH in the frail cohort. The disconnect reported previously between symptoms of OH and objective measures of orthostatic blood pressure may reflect that in some individuals with chronic hypotension it becomes somewhat well tolerated without overt symptoms, but may nevertheless be detrimental and impair cognitive function [51].

Given the previously reported associations between OH and cognitive impairment [50] we considered whether the incidence of deficits associated with cognitive symptoms was greater in those with OH than those without OH. We found that the incidence of Consensus OH (the most frequently reported sub-type of OH) was not greater in those with cognitive symptoms than those without. While this differs from previous associations, this does not necessarily mean that OH has no effect on cognition, merely that when cognitive disorders are considered as “present” or “absent” according to the concept of deficit accumulation, there is no distinction between OH subgroups; we may have observed a difference if the *severity* of the cognitive impairment were evaluated in the context of OH.

In the present study, both Initial and Consensus blood pressure responses were associated with frailty, but not Delayed responses. This might suggest that frail individuals are at particular risk for OH and associated falls in the first minutes after posture change or transfer, and suggests that care home staff pay particular attention to risk mitigation for frail older adults as they perform transfers or change posture. Another possibility is that Delayed OH is common among all older adults in long term care (49%), resulting in the lack of a difference between frail and non-frail groups. Further investigation could help elucidate the relationship between Delayed OH and frailty in older adults.

In frail older adults, there may be particular benefit to screening for OH, using this simple seated orthostatic stress test. The results of this test may be a warning sign for more conservative management of OH, such as medication adjustment, maintenance of adequate hydration, and sleeping with the head of the bed elevated [52]. Risks of falling increase in those with gait disturbance, poor balance, cognitive impairments, multiple comorbidities, and polypharmacy. These risk factors are associated with OH and are criteria often used to derive many FI [32]. This simple test could accompany the existing FI and provide further risk stratification of patients. Older adults who are both frail and exhibit impaired orthostatic blood pressure control are at particular risk for future falls and should receive tailored management accordingly.

For those who are not able to complete an orthostatic stress test, the FI may provide a modest surrogate marker for susceptibility to OH, whereby those with a FI ≥ 0.27 were at higher risk of experiencing both Initial and Consensus OH.

### Relationships between frailty and falling

We showed that individuals who were frail were more likely to have fallen in the past year, and went on to have more falls in the subsequent year. In a mixed sample of community dwelling and older adults resident in long term care in Canada, the FI was also correlated with future falls (r = 0.12; *p* < 0.001) [53]. In the current study of elderly residents in long term care facilities, the correlation between FI-MDS and prospective falls was slightly stronger (r = 0.21, *p* = 0.03), perhaps reflecting the higher risk of falls and frailty in general in this cohort.

We showed that prospective falling could be quite reliably predicted from a linear combination of FI-MDS and retrospective falling incidence (r = 0.550, *p* < 0.001). Further studies should evaluate the usefulness of the combination of FI-MDS and retrospective fall data to predict prospective falls.

ROC analyses showed that FI-MDS predicts future falls with reasonable sensitivity (~ 80%), but poor specificity. These results are comparable to other ROC analysis examining the utility of a FI to predict future falls in community dwelling adults [54]. Accordingly, use of the FI-MDS could be a useful simple screening tool to identify those who are at risk of future falls (with a FI ≥0.27), with the caveat that it would be less effective for excluding those who are not likely to experience future falls. This could be followed up with additional evaluation, to better identify those who are less likely to experience future falls [55]. As noted previously, older adults who are both frail and exhibit impaired orthostatic blood pressure control are at particular risk for future falls and should receive tailored management to mitigate this risk.

### Relationships between frailty and mortality

We have shown that the presence of frailty (FI-MDS ≥ 0.27) predicts 3-year mortality even in a cohort of individuals where the number of deficits and mortality rates are high. This is also seen in other studies, suggesting that the FI does not exhibit a ceiling effect for individuals living in long term care [56]. In community dwelling adults, the presence of frailty increases the risk of death at any age [57]. This can be observed even among cohorts with high levels of frailty, such as individuals living in long term residential care facilities [15, 44, 56–58]. ROC analyses showed that FI-MDS predicted 3-year mortality with reasonable sensitivity (77%), but poor specificity. These results are similar to previous reports using a FI to predict mortality within a 3 year follow up period [54] and further highlights the utility and robustness of the relationship between the FI-MDS and mortality among older care home residents, while recognising that mortality in general is high in this population. In the present study, the presence of prior falls was not significantly associated with mortality, while the presence of Consensus OH tended to be associated with higher mortality (*p* = 0.07). The lack of clear contribution to 3-year mortality from OH and falling may reflect the high levels and collinearity of OH (62%), falling (64%) and 3-year mortality (65%) in this cohort.

## Limitations

We used the MDS to derive the FI. While this approach was in keeping with the philosophy of previously validated frailty indices based on deficit accumulation [3, 10], and has been validated in the acute care hospital setting [18], the MDS was not designed for this purpose. Accordingly, while this does provide a convenient tool, the approach has not been fully validated. However, there is precedent for the use of routine data collection tools in older adults living in long term care homes to generate FI, with two validated FI developed from the Risk Assessment Index (RAI) [17] and the Complete Geriatric Assessment (CGA) [11]. Given that the mean FI derived from our FI-MDS was similar to other FI in home care populations, it is likely that this approach is suitable, but further validation of this method is needed.

The relatively small sample size in the present study, particularly for the OH measures, provides an obvious potential limitation impacting statistical power. Nevertheless, we were able to detect significant differences in orthostatic cardiovascular control between frail and non-frail individuals, and between fallers and non-fallers, as well as links to all-cause mortality, suggesting statistical confidence in our data. Future expansion of these observations to larger cohorts would be of benefit.

We did not include potential confounding factors in our analyses; however, they are incorporated within the frailty score by definition. These confounding factors would be considered as deficits according to specific criteria outlined previously [3] and so would be indirectly incorporated.

While we found significant relationships between frailty, falling, OH and mortality, it is likely that our measures of OH represent an underestimation of the true presence of OH in this cohort, because we evaluated OH during a relatively mild orthostatic stress, with passive seated orthostatic stress. The use of a standing posture might be expected to induce larger blood pressure declines and therefore reveal the presence of OH in more individuals. We opted to use seated orthostatic stress in this frail cohort because many participants would have found it difficult to complete a standing test. Furthermore, we showed previously that blood pressure responses to passive seated and standing orthostatic stresses are similar, at least for the first 8 min of assumption of the upright position [28], suggesting that our findings in the Initial and Consensus phases (first 3 min) are likely to be robust and reflective of the true incidence of OH in this cohort. One benefit of this approach is that a key factor associated with frailty and falling was the systolic blood pressure 1-min after the assumption of a passive seated position (Recovery OH). This test is simple, quick, practical and well-tolerated and could easily be incorporated into routine assessments. However, we accept that the inability to detect differences in the magnitude and prevalence of Delayed OH between frail and non-frail individuals could, at least in part, reflect the low level of orthostatic stress employed, because after 8 min responses to seated orthostatic stress are significantly smaller than during standing [28]. Accordingly, it is possible that the incidence and impact of Delayed OH in this cohort (49%) is an underestimate of the true scope of this issue.

In six individuals mortality data were not available because they were discharged from the facility to a higher level of care. In this cohort of individuals this is typically a herald of end of life care, and mortality occurs soon after discharge. We, therefore, included these individuals in our analysis as if the mortality end-point was met within one month of that time, which may not always have been the case. However, we do not believe this impacted our results, because when we repeated our analyses excluding these participants our results were unchanged.

## Conclusions

We have demonstrated that frailty can be captured using a validated frailty index [3] generated using MDS data in elderly individuals living in long term care, and is related to orthostatic cardiovascular control, falling risk and all-cause mortality. Frail individuals had larger Initial and Consensus orthostatic reductions in SAP, with impaired blood pressure recovery, a higher prevalence of OH and a higher mortality rate. Frail individuals also experienced higher prospective and retrospective rates of falling than the non-frail. Older adults who are both frail and have impaired orthostatic blood pressure control have a particularly high risk of falling and should receive tailored management to mitigate this risk. Use of the MDS to generate a frailty index may represent a simple and convenient tool for risk assessment of falling, OH and mortality in older adults living in long term care.

## Additional files

Additional file 1: *Full Resident Assessment Instrument - Minimum Data Set 2.0 coded into a frailty index (FI-MDS).* *Count if under dressing or personal hygiene but do not double count with P3g. ^‡^If either MDS category is present, score the deficit as 1. Abbreviations: ADL, activities of daily living; ALS, Amyotrophic Lateral Sclerosis; BMI, body mass index; MDS, minimum data set; MS, multiple sclerosis; FI, frailty index; COPD, chronic obstructive pulmonary disease. (DOCX 17 kb)

Additional file 2: *Domains of the Full Resident Assessment Instrument - Minimum Data Set 2.0 considered for generation of the frailty index (FI-MDS) (58 Items in total).* Each subdomain comprises related components of the MDS document. (DOCX 12 kb)

## Abbreviations

FI: derived frailty index
MDS: Minimum data set
AUC: area under curve
CGA: Comprehensive geriatric assessment
DAP: Diastolic arterial pressure
FI: Frailty index
MAP: Mean arterial pressure
MDS: minimum data set
OH: Orthostatic hypotension
RAI: Risk analysis index
ROC: receiver operating curve
SAP: Systolic arterial pressure

## References

[1] Vaupel JW (2010). Biodemography of human ageing. Nature..

[2] Rockwood K, Song X (2011). Changes in relative fitness and frailty across the adult lifespan: evidence from the Canadian National Population Health Survey. Can Med Assoc J [Internet].

[3] Rockwood K, Mitnitski A (2007). Frailty in relation to the accumulation of deficits. J Gerontol - Ser A Biol Sci Med Sci.

[4] Fried LP, Ferrucci L, Darer J, Williamson JD, Anderson G (2004). Untangling the concepts of disability, frailty, and comorbidity: implications for improved targeting and care. J Gerontol Ser A Biol Sci Med Sci [Internet].

[5] Gobbens RJ, Luijkx KG, Wijnen-Sponselee MT, Schols JM (2010). Toward a conceptual definition of frail community dwelling older people. Nurs Outlook [Internet].

[6] Cesari M, Prince M, Thiyagarajan JA, De Carvalho IA, Bernabei R, Chan P (2016). Frailty: an emerging public health priority. J Am Med Dir Assoc [Internet]..

[7] Schuurmans H, Steverink N, Lindenberg S, Frieswijk N, Slaets JPJ (2004). Old or frail: what tells us more?. J Gerontol Ser A Biol Sci Med Sci [Internet]..

[8] Kaethler Y, Molnar FJ, Mitchell SL, Soucie P, Man-Son-Hing M (2003). Defining the concept of frailty: a survey of multi-disciplinary health professionals. Geriatr Today J Can Geriatr Soc.

[9] Moorhouse P, Rockwood K (2013). Frailty and its quantitative clinical evaluation. J R Coll Physicians Edinb.

[10] Searle SD, Mitnitski A, Gahbauer EA, Gill TM, Rockwood K (2008). A standard procedure for creating a frailty index. BMC Geriatr.

[11] Jones DM, Song X, Rockwood K (2004). Operationalizing a frailty index from a standardized comprehensive geriatric assessment. J Am Geriatr Soc.

[12] Cesari M, Gambassi G, Van Kan GA, Vellas B (2014). The frailty phenotype and the frailty index: different instruments for different purposes. Age Ageing.

[13] Rockwood K (2016). Conceptual models of frailty: accumulation of deficits. Can J Cardiol.

[14] Fried LP, Tangen CM, Walston J, Newman AB, Hirsch C, Gottdiener J et al, Fried LP, Tangen CM, Walston J et al. frailty in older adults: evidence for a phenotype. J Gerontol A Biol Sci Med Sci 2001;56(3):M146–M156. 10.1093/gerona/56.3.m146 11253156

[15] Hoover M, Rotermann M, Sanmartin C, Bernier J. Article Validation of an index to estimate the prevalence of frailty among community -dwelling seniors 2013;24(82):10–17. Available from: http://www.statcan.gc.ca/pub/82-003-x/2013009/article/11864-eng.htm 24258362

[16] Mitnitski A, Fallah N, Rockwood K (2011). A multistate model of cognitive dynamics in relation to frailty in older adults. Ann Epidemiol [Internet].

[17] Hall DE, Arya S, Schmid KK, Blaser C, Carlson MA, Bailey TL (2017). Development and initial validation of the risk analysis index for measuring frailty in surgical populations. JAMA Surg.

[18] Hubbard RE, Peel NM, Samanta M, Gray LC, Fries BE, Mitnitski A (2015). Derivation of a frailty index from the interRAI acute care instrument. BMC Geriatr.

[19] The Consensus Committee of the American Autonomic Society and the American Academy of Neurology (1996). Consensus statement on the definition of orthostatic hypotension, pure autonomic failure, and multiple system atrophy. Neurology.

[20] Wieling W, Schatz IJ (2009). The consensus statement on the definition of orthostatic hypotension: a revisit after 13 years. J Hypertens.

[21] Varadhan R, Seplaki CL, Xue QL, Bandeen-Roche K, Fried LP (2008). Stimulus-response paradigm for characterizing the loss of resilience in homeostatic regulation associated with frailty. Mech Ageing Dev.

[22] O’Connell MDL, Savva GM, Fan CW, Kenny RA (2015). Orthostatic hypotension, orthostatic intolerance and frailty: the Irish longitudinal study on aging-TILDA. Arch Gerontol Geriatr [Internet].

[23] Theou O, Brothers TD, Mitnitski A, Rockwood K (2013). Operationalization of frailty using eight commonly used scales and comparison of their ability to predict all-cause mortality. J Am Geriatr Soc.

[24] Gill TM, Gahbauer EA, Han L, Allore HG (2010). Trajectories of disability in the last year of life. N Engl J Med [Internet].

[25] Rockwood MR, Howlett SE, Rockwood K. Orthostatic hypotension (OH) and mortality in relation to age, blood pressure and frailty. Arch Gerontol Geriatr. 2012;54(3):e255-60. 10.1016/j.archger.2011.12.009 22240412

[26] Romero-Ortuno R, Cogan L, Foran T, Kenny RA, Fan CW (2011). Continuous noninvasive orthostatic blood pressure measurements and their relationship with orthostatic intolerance, falls, and frailty in older people. J Am Geriatr Soc.

[27] Lagro J, Schoon Y, Heerts I, ASS M-VDA, Schalk B, Wieling W (2014). Impaired systolic blood pressure recovery directly after standing predicts mortality in older falls clinic patients. Journals Gerontol - Ser A Biol Sci Med Sci..

[28] Shaw BH, Loughin TM, Mackey DC, Robinovitch SN, Claydon VE. The effect of orthostatic stress type on cardiovascular control. Blood Press Monit [Internet]. 2014;19(6):327–38 Available from: https://journals.lww.com/bpmonitoring/pages/articleviewer.aspx?year=2014&issue=12000&article=00003&type=abstract. 10.1097/MBP.0000000000000067 25121755

[29] Pasma JH, Bijlsma AY, Klip JM, Stijntjes M, Blauw GJ, et al. Blood Pressure Associates with Standing Balance in Elderly Outpatients. PLoS ONE. 2014;9(9):e106808. 10.1371/journal.pone.0106808 PMC4164445 25222275

[30] Robinovitch SN, Feldman F, Yang Y, Schonnop R, Leung PM, Sarraf T (2013). Video capture of the circumstances of falls in elderly people residing in long-term care: an observational study. Lancet [Internet].

[31] World Health Organization. WHO Global Report on Falls Prevention in Older Age. Community Health (Bristol) [Internet]. 2007;53. Available from: http://www.who.int/ageing/publications/Falls_prevention7March.pdf

[32] Shaw BH, Claydon VE (2014). The relationship between orthostatic hypotension and falling in older adults. Clin Auton Res.

[33] Rubenstein LZ (2006). Falls in older people: epidemiology, risk factors and strategies for prevention. Age Ageing.

[34] Rubenstein LZ, Josephson KR, Osterweil D (1996). Falls and fall prevention in the nursing home. Clin Geriatr Med.

[35] Iwanczyk L, Weintraub NT, Rubenstein LZ (2006). Orthostatic hypotension in the nursing home setting. J Am Med Dir Assoc.

[36] Ravensbergen HRJC, Mcgrath MS, Claydon VE. Cerebrovascular Responses to Orthostatic Stress after Spinal Cord Injury 2012;2456:2446–2456. 10.1089/neu.2012.2379 22720841

[37] Freeman R, Wieling W, Axelrod FB, Benditt DG, Benarroch E, Biaggioni I (2011). Consensus statement on the definition of orthostatic hypotension, neurally mediated syncope and the postural tachycardia syndrome. Clin Auton Res.

[38] Heitterachi E, Lord SR, Meyerkort P, McCloskey I, Fitzpatrick R (2002). Blood pressure changes on upright tilting predict falls in older people. Age Ageing.

[39] Romero-Ortuno R, Cogan L, Fan CW, Kenny RA (2010). Intolerance to initial orthostasis relates to systolic BP changes in elders. Clin Auton Res.

[40] Hutchinson AM, Milke DL, Maisey S, Johnson C, Squires JE, Teare G, et al. The Resident Assessment Instrument-Minimum Data Set 2. Psychiatr Nurses Assoc. 2012;18(2 SRC-GoogleScholar FG-0):1472–6963. 10.1186/1472-6963-10-166 PMC2914032 20550719

[41] Scott V, Peck S, Kendall P. Prevention of Falls and Injuries Among the Elderly [Internet]. Health (San Francisco). 2004. 96 p. Available from: https://www2.gov.bc.ca/gov/content/health/about-bc-s-health-care-system/office-of-the-provincial-health-officer/reports-publications/special-reports

[42] Kojima G, Kendrick D, Skelton DA, Morris RW, Gawler S, Iliffe S (2015). Frailty predicts short-term incidence of future falls among British community-dwelling older people: a prospective cohort study nested within a randomised controlled trial physical functioning, physical health and activity. BMC Geriatr [Internet].

[43] Ludwig C, Busnel C (2017). Derivation of a frailty index from the resident assessment instrument - home care adapted for Switzerland: a study based on retrospective data analysis. BMC Geriatr.

[44] Theou O, Tan ECK, Bell JS, Emery T, Robson L, Morley JE (2016). Frailty levels in residential aged care facilities measured using the frailty index and FRAIL-NH scale. J Am Geriatr Soc.

[45] Doorn C Van, Gruber-baldini AL, Zimmerman S, Hebel JR, Port CL, Baumgarten M, et al. Nursing Home Residents 2003;1213–1218. 10.1046/j.1532-5415.2003.51404.x 12919232

[46] Mitnitski A, Song X, Skoog I, Broe GA, Cox JL, Grunfeld E (2005). Relative fitness and frailty of elderly men and women in developed countries and their relationship with mortality. J Am Geriatr Soc.

[47] Tabue-Teguo M, Kelaiditi E, Demougeot L, Dartigues JF, Vellas B, Cesari M (2015). Frailty index and mortality in nursing home residents in France: results from the INCUR study. J Am Med Dir Assoc [Internet].

[48] Rockwood K, Andrew M, Mitnitski A (2007). A comparison of two approaches to measuring frailty in elderly people. J Gerontol a biol Sci med Sci [internet].

[49] Gupta V, Lipsitz LA (2007). Orthostatic hypotension in the elderly: diagnosis and treatment. Am J Med.

[50] Mehrabian S, Duron E, Labouree F, Rollot F, Bune A, Traykov L (2010). Relationship between orthostatic hypotension and cognitive impairment in the elderly. J Neurol Sci [Internet].

[51] Jegede AB, Rosado-Rivera D, Bauman WA, Cardozo CP, Sano M, Moyer JM (2010). Cognitive performance in hypotensive persons with spinal cord injury. Clin Auton Res [Internet].

[52] Moya A, Sutton R, Ammirati F, Blanc JJ, Brignole M, Dahm JB (2009). Guidelines for the diagnosis and management of syncope (version 2009). Eur Heart J.

[53] Kanters DM, Griffith LE, Hogan DB, Richardson J, Patterson C, Raina P (2017). Assessing the measurement properties of a frailty index across the age spectrum in the Canadian longitudinal study on aging. J Epidemiol Community Health.

[54] Li G, Thabane L, Ioannidis G, Kennedy C, Papaioannou A, Adachi JD (2015). Comparison between frailty index of deficit accumulation and phenotypic model to predict risk of falls: data from the global longitudinal study of osteoporosis in women (GLOW) Hamilton cohort. PLoS One.

[55] Lalkhen AG, McCluskey A (2008). Clinical tests: sensitivity and specificity. Contin Educ Anaesthesia, Crit Care Pain.

[56] Ridda I, Lindley R, MacIntyre RC. The challenges of clinical trials in the exclusion zone: The case of the frail elderly. Australas J Ageing. 2008;27(2):61–6. 10.1111/j.1741-6612.2008.00288.x 18713194

[57] Song X, Mitnitski A, Rockwood K. Prevalence and 10-Year outcomes of frailty in older adults in relation to deficit accumulation. J Am Geriatr Soc. 2010;58(4):681–7. 10.1111/j.1532-5415.2010.02764.x 20345864

[58] Rockwood K, Abeysundera MJ, Mitnitski A. How should we grade frailty in nursing home patients? J Am Med Dir Assoc. 2007;8(9):595–603. 10.1016/j.jamda.2007.07.012 17998116

