# Supplementary material for: Relationships between orthostatic hypotension, frailty, falling and mortality in elderly care home residents
Source: BMC Geriatr. 2019 Mar 13;19:80. doi: 10.1186/s12877-019-1082-6 (PMC6415493; doi:10.1186/s12877-019-1082-6)
Supplement: Supplementary file 1 — Full Resident Assessment Instrument - Minimum Data Set 2.0 coded into a frailty index (FI-MDS). *Count if under dressing or personal hygiene but do not double count with P3g. ‡If either MDS category is present, score the deficit as 1. Abbreviations: ADL, activities of daily living; ALS, Amyotrophic Lateral Sclerosis; BMI, body mass index; MDS, minimum data set; MS, multiple sclerosis; FI, frailty index; COPD, chronic obstructive pulmonary disease. (DOCX 17 kb) [file 12877_2019_1082_MOESM1_ESM.docx]

| **Deficit (58 Items)** | **MDS Category** | **MDS Section** | **Score** |
| --- | --- | --- | --- |
| Short Term Memory Impairment | Memory - Short Term Memory Impairment | B2:a | No = 0, Yes = 1 |
| Long Term Memory Impairment | Memory - Long Term Memory Impairment | B2:b | No = 0, Yes = 1 |
| Memory Changes | Memory / Recall ability | B3 | None present = 1  At least 1 present = 0 |
| Onset of cognitive symptoms | Cognitive skills for daily decision making  Changes in cognitive status | B4  B6 | 0 = 0, 1/2/3 = 1^‡^  0 = 0, 1 = 0, 2 = 1^‡^ |
| Changes in general mental functioning | Indicators of delirium, disordered thinking/awareness  Alzheimer’s or Dementia special care unit | B5: a, b, c, e, f  P1a: n | Not Present = 0  At least 1 present = 1 |
| Clouding or Delirium | Delusions, Hallucinations | J1e, i | Not present = 0  At least 1 present = 1 |
| Restlessness | Periods of restlessness  Repetitive physical movements | B5: d  E1: n | Not present = 0  At least 1 present = 1 |
| Change in communication | Change in communication/ hearing | C7 | 0 = 0, 1 = 0, and 2 = 1 |
| Feeling sad, blue, depressed | Negative statements, repetitive verbalization, self deprecation | E1: a, c, e, m | Not present = 0  At least 1 present = 1 |
| Paranoid features | Expression of unrealistic fears, recurrent statements that something terrible will happen. | E1: f, g | Not present = 0  At least 1 present = 1 |
| Anxiety | antianxiety drugs, repetitive anxious complaints | O4: b  E1: i | 0 = 0, 1/2 = 1^‡^  At least 1 day = 1^‡^ |
| Sleep changes | Insomnia or change in sleep patterns | E1: k | 0 = 0, 1/2 = 1 |
| Social interaction | Reduced social interaction  At ease interacting with others | E1p  F1a | 0 = 0, 1/2 = 1^‡^  Not Marked = 0, Marked = 1^‡^ |
| Reduction in usual activity | Loss of interest: withdrawn from activities of interest | E1: o | No = 0, Yes = 1 |
| Mood problems | Mood persistence | E2 | 0 = 0, 1/2 = 1 |
| Having trouble getting going | At ease doing self initiated activities | F1: c | Marked = 0, Not marked = 1 |
| Impaired mobility | Bed mobility, walk in room, walk in corridor, locomotion on unit, locomotion off unit  Modes of transfer (bedfast, bed rails)  Bed mobility, transfer, walking rehab program | G1: a, c, d, e, f  G6  P3d, e, f | 0 = 0, 1/2/3/4/8 = 1^‡^  At least 1 present = 1^‡^  At least 1 day = 1^‡^ |
| Changes in everyday activities | ADL self performance | G9 | (0 = 0, 1 = 0, and 2 = 1) |
| Problems getting dressed (graded score) | Dressing | G1g  P3g* | 0 = 0, 1/2/3/4/8 = 1^‡^  At least 1 day = 1^‡^ |
| Toileting problems | Toileting use | G1i | 0 = 0, 1/2/3/4/8 = 1 |
| Personal hygiene and grooming | Grooming | G1j + p3g* | 0 = 0, 1/2/3/4/8 = 1  At least 1 present = 1 |
| Problems with bathing | Bathing | G2 | 0/1 = 0, 2/3/4/8 = 1 |
| Poor standing posture | Balance while standing | G3: a | 0 = 0, 1/2/3 = 1 |
| Poor coordination, trunk | Balance while sitting | G3: b | 0 = 0, 1/2/3 = 1 |
| Functional limitation in range of motion | Functional limitation in range of motion | G4A: b, c, d, e, f | Not present = 0  At least 1 present = 1 |
| Head and neck problems | Functional limitation in range of motion and voluntary movement | G4A/B: a | Not present = 0  At least 1 present = 1 |
| Functional limitation in voluntary movement | Functional limitation in voluntarily movement | G4B: b, c, d, e, f, | Not present = 0  At least 1 present = 1 |
| Help getting in and out of chair | Transfer | G1b A/B | 0 = 0, 1/2/3/4/8 = 1 |
| Help eating | Eating  Eating or swallowing  Oral problems  Feeding tube | P3h  G1A/B: h  K1  K5: b | Not present = 0  At least 1 present = 1 |
| Nutritional problems | Nutritional problems and approaches | K4c  K5 | Not present = 0  At least 1 present = 1 |
| Urinary incontinence | Control of urinary bladder function  Appliances and programs | H1b  H3 | 0 = 0, 1/2/3/4 = 1^‡^  At least 1 present = 1^‡^ |
| Bulk difficulties | Constipation | H2: d | No = 0, Yes = 1 |
| Gastrointestinal problems | Gastrointestinal disease  Diarrhea | I1: ss  H2c | Not present = 0  At least 1 present = 1 |
| Bowel incontinence | Bowel continence | H1a | 0 = 0, 1/2/3/4 = 1 |
| History of diabetes mellitus | Diabetes mellitus | I1: a | No = 0, Yes = 1 |
| Musculoskeletal problems | Hip fractures | I1: m | No = 0, Yes = 1 |
| History of degenerative disease | ALS, dementia other than Alzheimer’s disease, Huntington’s disease, MS  Cancer, Diabetes | I1q, I1v, I1x, I1y  P1a: a, h | Not present = 0  At least 1 present = 1 |
| History of stroke | Cerebrovascular accident (stroke) | I1u | No = 0, Yes = 1 |
| Depression (clinical depression) | Depression  Antidepressant drugs | I1gg  O4c | Not present = 0  At least 1 day = 1 |
| Other psychiatric illnesses | Psychological therapy, Bipolar disorder, Schizophrenia  Antipsychotic drugs | P1b: e, I1hh, ii  O4: a | Not present = 0  At least 1 present = 1^‡^  At least 1 day = 1^‡^ |
| Other medical history | Disease diagnoses (if not counted before), infections not included | I1, J1f, g, j, o | Not present = 0  At least 1 present = 1 |
| Cancer | Cancer  Radiation, chemotherapy | I1: rr  P1a: a, h | No = 0, Yes = 1  At least 1 present = 1 |
| Lung problems | Recurrent lung aspirations in last 90 days, shortness of breath  Pneumonia and respiratory infections | J1: k, l  I2g, I2f | Not present = 0  At least 1 present = 1 |
| High blood pressure | Benign hypertension | I3b (Quarterly Assessment) | Not present = 0  At least 1 present = 1 |
| Irregular gait patterns | Unsteady gait | J1: n | No = 0, Yes = 1 |
| Syncope or blackouts | Syncope (fainting) | J1: m | No = 0, Yes = 1 |
| Sense of pain | Pain | J2: a | 0 = 0, 1/2 = 1 |
| History of falls | Accidents | J4 | Not present = 0  At least 1 present = 1 |
| BMI | Height and weight | K2 | For male and female < 18.5, ≥ 30 as deficit |
| Weight loss in last 30 days | Weight change | K3 | No = 0, Yes = 1 |
| Skin problems | Ulcer, other problems, or skin treatment | M2, M4, M5 | Not present = 0  At least 1 present = 1 |
| Medications | Number of medications | O1 | >5 = 1, >10 = 2, >15 = 3, >20 = 4 |
| Difficulty speaking or communicating | Making self understood, Ability to understand others  Communication training | C4, C6  P3j | No = 0, Yes = 1^‡^  At least 1 day = 1^‡^ |
| History of renal failure | Renal failure, renal dialysis | I1uu, P1a: b | Not present = 0  At least 1 present = 1 |
| Respiratory problems | Asthma, COPD  Oxygen therapy, suctioning, ventilator or respirator use, tracheostomy care  Speech – Language pathology, Audiology service, Respiratory therapy | I1: jj, kk  P1a: g, i, l, j  P1b: a, d | Not present = 0  At least 1 present = 1  At least 1 day = 1 |
| Alcohol use | Alcohol or drug treatment program | P1a: m | No = 0, Yes = 1 |
| History of hypo/hypertension | History of hypertension | I1: h, I | No = 0, Yes = 1 |
| History of Thyroid disease | History of thyroid disease | I1: b, c | No = 0, Yes = 1 |
