# Supplementary material for: Relationships between orthostatic hypotension, frailty, falling and mortality in elderly care home residents
Source: BMC Geriatr. 2019 Mar 13;19:80. doi: 10.1186/s12877-019-1082-6 (PMC6415493; doi:10.1186/s12877-019-1082-6)
Supplement: Supplementary file 2 — Domains of the Full Resident Assessment Instrument - Minimum Data Set 2.0 considered for generation of the frailty index (FI-MDS) (58 Items in total). Each subdomain comprises related components of the MDS document. (DOCX 12 kb) [file 12877_2019_1082_MOESM2_ESM.docx]

| **Physical Function** | **ADL** | **Mobility and Balance** |
| --- | --- | --- |
| Restlessness  Reduction in usual activity  Having trouble getting going  Head and neck problems  Sense of pain  Alcohol use | Sleep changes  Changes in every day activities  Problems getting dressed  Toileting problems  Personal hygiene and grooming  Problems with bathing  Help eating  Nutritional problems | Impaired mobility  Poor coordination, trunk  Functional limitation in range of motion  Functional limitation in voluntary movement  Help getting in and out of chair  Irregular gait patterns  History of falls  Poor standing posture  Restlessness |
| **Medical Problems** | **Chronic Disease** | **Cognition – Non-Dementia** |
| Gastrointestinal problems  Musculoskeletal problems  Other medical history  Skin problems | Cancer  History of diabetes mellitus  History of degenerative disease  History of thyroid disease | Short-term memory impairment  Long-term memory impairment  Changes in general mental functioning  Onset of cognitive symptoms  Memory changes |
| **Psychiatric Illness** | **Bladder and Bowel** | **Cardiovascular Disease** |
| Paranoid features  Anxiety  Other psychiatric illnesses  Clouding or delirium | Urinary incontinence  History of renal failure  Bulk difficulties  Bowel incontinence | History of stroke  Syncope or blackouts  High blood pressure  History of hypo/hypertension |
| **Social** | **Respiratory Disease** | **Mood** |
| Change in communication  Difficulty speaking or communicating  Social interaction | Lung problems  Respiratory problems | Feeling sad, blue depressed  Depression  Mood problems |
